# Vaccination in the Light of the Royal British Commission

**Published:** 1897-04

**Authors:** M. R. Leverson

**Affiliations:** Fort Hamilton, L. I.


### VACCINATION IN THE LIGHT OF THE ROYAL BRITISH COMMISSION.

M. R. LEVERSON, M. D., FORT HAMILTON, L. I.

(Continued from page 85.)

Dr. Arthur F. Hopkirk referred for his authority with regard to Prussia to a paper by Dr. Guttstadt, in the *Zeitschrift des Koniglichen Preussichen Statistischen Bureaus* for 1873, which he stated is a complete report upon the epidemic of 1870-71, and of all the epidemics from the end of the last century to the end of 1872 (Q. 1,431, *Second Rep. Com. of* 1889, p. 8a). In Q. 1,433 he speaks thus of the vaccination laws of Prussia: "That is a point upon which there has been a good deal of misunderstanding in England. In 1834 a law was passed making vaccination and re-vaccination compulsory for all the soldiers in the Prussian army; *but there was no law making vaccination itself compulsory for the civil population of Prussia until the 8th of April,* 1874, a law which came into action in 1875."

In Q. 1,434 he says: "Every German authority at the present day states that there was no real compulsion until 1874."

This was stated on the 9th of October, 1889. Dr. Hopkirk made other remarkable statements at this time, which will be noticed later. Inquiries being made by the British Government at the request of the commission, of the German (and French) governments with reference thereto, the replies received did not at all agree with Dr. Hopkirk's statements. He was therefore recalled before the commission on the 19th of February, 1890. The value of Dr. Hopkirk's statements will be more readily appreciated by presenting the contradictions of the 19th of February, 1890, to the statements of the 9th of October, 1889, immediately after the latter; accordingly, we now give Q. 6,792, page 222 b, same report.

Dr. Hopkirk being a good German scholar, he was required to read and translate the law of 1835. "I have approved these rules and now confirm them with an order that they should be obeyed by every one within the whole extent of my dominion, under penalty of the fine and imprisonment provided; and that all officials concerned should act upon them." Dr. Hopkirk, who on the 9th of October, 1889, had stated "there was no law making vaccination compulsory for the civil population of Prussia," now says: "I cannot say that I ever studied that enactment of 1835."

Among the "rules" thus "confirmed" and ordered to be obeyed within the whole extent of the Prussian dominions was the following: "Heads of schools, master craftsmen, tradesmen, and the employer of servants will do well to satisfy themselves that those who have entered on a course of instruction with them, or into apprenticeship, or have taken service with them, have been vaccinated; persons who seek any scholarship or other benefices for their children or their wards, or who seek to place them in any public establishment, are to be refused if they cannot furnish proof of their vaccination having been duly performed." (Q. 6,791.) Perhaps I may be excused for here interpolating some personal experience. In 1866 I placed my four children at school in Gottingen (Province of Hanover) and before they were admitted proof had to be given that they had been vaccinated, and such of them as did not present satisfactory "marks" had to be re-vacccinated. In 1872, when I removed them to the city of Hanover, the like evidence had to be repeated. In 1875, I being then forty-five years of age, wished to "hospitiren," or, as it is termed in the United States, take a special course, in assaying at the Clausthal Mining School; but before I could be admitted I had to prove either that I had been vaccinated or had had the small-pox. Having been vaccinated at the age of two years and had confluent small-pox at three, I was able to give evidence of both. Let us return to Dr. Hopkirk on the 9th of October, 1889. He gives a large number of statistics (Q. 1,463), which even if accurate have no bearing on the subject, for while they show, *for a time,* a decrease of small-pox deaths following the introduction of vaccination, yet as observed by Dr. Farr this decrease commenced *before* its introduction. Small-pox was an epidemic disease and wherever conditons of filth prevails it is so still; but because it is epidemic the mortality it causes year by year rises and falls, and as is even shown very clearly by Dr. Hopkirk's "Diagram Showing the Mortality from Small-pox in Prussia from 1816 to 1886 per 100,000 inhabitants." (App. No. 2, to face p. 232 of the second report of the commission) of all the epidemics of the century, beginning with that of 1816, that of 1871 and 1872 was the worst, there having died in each of those years in Prussia nearly four times as many per 100,000 living as in the next most fatal epidemic—that of 1866.

Q. 1,467, quoting Lotz's *Pocken und Vaccination,* which Dr. Hopkirk claims to have carefully verified, he says: "He shows how high the mortality was amongst young children up to the age of twenty in the days before vaccination, while after the age of twenty *there was scarcely anybody left to have small-pox.* They had all had it; they *had either died from it or had been disfigured by it.*"

The absurdity of this statement even when inoculation was at its worst, is too palpable to need refutation. As shown above (p. 499, Vol. XVI), with reference to a similar statement by Sir John Simon with regard to England and Wales, had it been true, Prussia would have been depopulated in a very short period. In Q. 1,467 additional evidence is there given tending to show that small-pox was formerly much more of an infantile disease than it is at present; other statistics point to the same conclusion. The advocates of vaccination claim this as evidence that the practice is beneficial. Yet, if it be in any way connected therewith, instead of an advantage it must be one of its serious evils. If out of a given number of births a certain number are to die of small-pox before they attain the age of twenty years, it is clearly far better that they should die in infancy than in youth. Not only has there been a less material expenditure upon them, but the mental suffering is also less than when the child has grown into the affections of its parents by the interchange of kindliness between parent and child through a term of years during which habit and use serve to reinforce mere animal love and to make the parting more painful when it comes. From an economic point of view, also, the loss is less to the community when the child dies in infancy than when it has begun to be a factor in production. But, further, vitality and the power of resistance to disease are far greater in youth and young adults than in infancy, and to assert that small-pox has now become a disease of the stronger life instead of the weaker, as it formerly was, proves, *if it be true*, that it has become a more serious disease than when Sydenham, "the great physician," "the English Hippocrates," "the father of modern medicine" (for by all these names is he known), declared small-pox to be "the lightest and safest of all diseases." Especially must this be true when it is found, as the fact is, that the fatality of small-pox is about the same (in old-school practice) to-day as it was 100 years ago. Under homeopathic and hydropathic treatment the fatality is somewhat less; but as all hydropathic physicians and most of the homeopathic condemn vaccination, the diminished fatality which attends the practice of physicians of these schools can afford but poor encouragement to the vaccinists.

The real bearings of a deferred age incidence of small-pox do not seem to have been recognized by those who have advanced the postponement of the age incidence as an effect of vaccination, or there is much reason to believe from their conduct, with relation to other aspects of the question, that they would have been anxious to suppress the facts instead of bringing them forward. Their having brought forward this change in age incidence as a proof of the benefits of vaccination serves to illustrate the inveteracy of the prejudices which blind many of the advocates of the rite to the most palpable truths. The evidence with regard to the change in age incidence of small-pox already obtained is strong, but not yet conclusive. If it were, as it would still, for the most part, be of the *post hoc ergo propter hoc* order, I should hesitate to claim it as an additional proof of the mischief of vaccination because of the total irrelevancy of the pathology of cow-pox to that of small-pox.

On the other hand, some of the many sources of vaccine matter in use have been obtained by inoculating the cow with the small-pox poison, so that vaccination, where this poison has been employed, is really variolation plus an unknown quantity of cow disease. Where, then, the poison used has been thus obtained the evidence that the postponement of the age incidence may be a result of this method of blood poisoning is more direct and becomes forcible.

Returning to Dr. Hopkirk's testimony, Q. 1,468 puts in very elaborate tables from which (Q. 1,469) Dr. Hopkirk drew the conclusion that in the epidemic of 1870—in Chemnitz—not a single vaccinated child under ten years died, but that of the unvaccinated a good many did. He also says that "All the children who died were unvaccinated and of the adults three males and four females were vaccinated, the remainder were unvaccinated." He also introduced statistics relative to other German towns, and asserted that in Russia also "none of the children under ten died who were vaccinated." He gave Dr. Guttstadt as his authority for this statement. He also (Q. 1,471) made a comparison (table F, App. II, p. 236, of second report of commission) between the mortality from small-pox in three small towns of Bavaria, Rawics, Bojanowo, and Sacruowo, from December,

1795, to the end of 1796, and that of Leipzig in 1871, and another table (Table G, App. II, p. 237) showing a comparison between the mortality from small-pox in those same towns in the same year and the mortality from small-pox in Lower Franconia for 1866-7, "primary vaccination being compulsory in the latter instance."

In Q. 1,472 he gives a calculation of the mortality from small-pox per 100,000 living in the three towns before named in a pre-vaccination year (1795-6), viz., 149.2, and contrasts it with that of Lower Franconia in 1866-7, with primary vaccination only, and a mortality of 28.6 per 100,000 living, each being an epidemic year; but he took no heed of the fact that in the former year inoculation was rampant, and that many if not all cases of death from measles were almost certainly included in the small-pox returns, also that there is a very great variation in the intensity of epidemics of small-pox as of other diseases. His Chemnitz statistics must be relegated to the region of the fabulous; not only are they absurd in themselves, but his like assertions with regard to the safety of children in Prussia having been capable of verification were tested and proved to be untrue, as presently shown.

But, again, his Chemnitz table (Table E, App. No. 2, second report, p. 236) gives 224 cases admitted to the hospital, 184 were vaccinated and only 37 alleged to be unvaccinated (Q. 1,602); yet he says (1,603) out of 3,546 cases in the town 2,643 were unvaccinated, and that, too, in the face of compulsory vaccination. Of the 249 deaths in Chemnitz 242 were reported as unvaccinated, yet in Berlin from 1871 to 1872 there were 4,524 deaths and only 1,383 alleged to be unvaccinated (Q. 1,596). In (Q. 1,649) he gives (Table E) the fatality among the unvaccinated in Chemnitz at nine per cent., and of the vaccinated in Coblenz eighteen per cent. of all ages (Table N).

In Q. 1,489 Dr. Hopkirk attempts to explain away the fact that of 30,742 cases of small-pox in Bavaria in 1871 20,429, or 95.7 per cent., were those of vaccinated persons, and only 1,313, or 4.3 per cent., were unvaccinated. His explanation is more ingenious than ingenuous. It is as follows: "If during so mighty an invasion of small-pox as that which crossed into and over Germany from France in the hard times of the war, 1871, only .68 per cent. of the entire Bavarian population suffered from the disease, then one is thoroughly justified in assuming that this population which was only so slightly attacked was afforded some special protection, and one may well ask how it would have been with the Bavarian population if it had not possessed this protection?"

But of what use is a "protection" which fails to protect at the only time that "protection" is needed? Small-pox is, as it always has been and always will be until extinguished by sanitation, an epidemic disease, and we are to be protected against this epidemic condition by a nostrum which is of no use in the face of an epidemic! But, further, an attack rate of .68 per cent. of the entire population in a single year is by no means an insignificant percentage; it is more than the attack rate in Great Britain during either 1871 or 1872, and nearly equal to that of those two years combined; it is greater than the attack rate in London in any year during this century.

At Q. 1,543 Dr. Hopkirk gives the now famous statement that of the French army in the Franco-Prussian war 23,409 died of small-pox in 1871, and of the Prussian army 316, and he claims these to be official statistics. As vaccination had been enforced upon every recruit in the French army for many years it is hard to see how this could tell in favor of vaccination even if it were true.

But the truth is that these figures are all imaginary—"evolved from the inner consciousness" of some one. The figures, or something like them, were first given to the English public by Dr. W. B. Carpenter in a letter to the members of the British Parliament. Mr. Alex. Wheeler, of Darlington, England, thereupon wrote to the war office in Paris and received the following reply:

Ministere de la Guerre,
Paris, 2 Juillet, 1883.

Monsieur:—En reponse a votre lettre du six Juin dernier j'ai l'honneur de vous faire savoir que nos ne possedons pas les chiffres des morts par suite de variole pendant la periode de la guerre de 1870-71, les statistiques medicales de ces annees n'ayant point pu être recueillies. Recevez, monsieur, l'assurance de ma consideration distinguée.

Pour le Ministre et par son ordre le medecin Inspecteur Directeur.

S. MILIOR, *Directeur.*

A. MONSIEUR WHEELER, Darlington.

Mr. Wheeler called on Dr. Carpenter to withdraw his statement. After some time, which it may be supposed he occupied in making inquiries which he ought to have made at first, Dr. W. B. Carpenter in August, 1883, published in the *London Daily News* a letter to the effect of the statement of the French Minister of War and withdrew his figures.

On the falsehood being rehashed by Dr. Hopkirk fresh inquiries were made with the same results.

Dr. Hopkirk testified (Q. 1,543) that the above statement had been recently confirmed from Paris, and that he "believed the recent confirmation was official," which statement he repeated when recalled (Q. 6,774), adding "that it is an absolute fact." He was then made to translate from the annual reports presented to the Minister of the Interior (from which he had himself quoted in his answer) (Q. 1,546) the mortality from small-pox in the French army, the deaths in 1863 "are given as 67; for 1864, 69; for 1865, 55; for 1866, 43; for 1867, 70; for 1868, 156; for 1869, 63; for 1870 and 1871, *no statistics.*"

The same was found to be the case with regard to the alleged German mortality from small-pox, viz., *that there was no possibility of telling the number.* That among a defeated, demoralized French army, among whom no attempt was made to secure cleanliness, personal or general, the mortality from small-pox as well as from other camp diseases was much higher than among the victorious, elated Germans, among whom the highest state of discipline prevailed throughout the war, "goes without saying." There is absolutely no evidence whatever to support the assertion that vaccination served to protect either army.

